# Supplementary material for: Locomotion and Postural Control in Young Adults with Autism Spectrum Disorders: A Novel Kinesiological Assessment
Source: J Funct Morphol Kinesiol. 2024 Oct 3;9(4):185. doi: 10.3390/jfmk9040185 (PMC11503382; doi:10.3390/jfmk9040185)
Supplement: Supplementary file 1 [file jfmk-09-00185-s001.zip › jfmk-3207930-supplementary.pdf]

Locomotion and Postural Control in Young Adults with Autism Spectrum Disorders: A Novel Kinesiological Assessment

Riccardo Di Giminiani [1,*], Stefano La Greca[1], Stefano Marinelli[1], Margherita Attanasio[1], Francesco Masedu[1], Monica Mazza[1], Marco Valenti[1]

1. Department of Biotechnological and Applied Clinical Sciences, University of L'Aquila, Italy

Supplementary Materials

List of content:

- **Table S1** Body sway values measured during static upright in ASD and TD. Median values and interquartile range are reported.
- **Table S2** Body sway values measured during dynamic upright standing in ASD and TD. Median values and interquartile range are reported.
- **Table S3** Surface electromyographic (sEMG) activity of leg muscles synchronized with body sway during static upright standing. Median values and interquartile range are reported
- **Table S4** Surface electromyographic (sEMG) activity of leg muscles synchronized with body sway during dynamic upright standing. Median values and interquartile range are reported.

Table S1. Body sway values measured during static upright in ASD and TD. Median values (interquartile range) are reported.

Variables	ASD	TD	p-Values
**BPS-CE-RL**	393.1(321.0-447.6)	472.3(410.8-533.5)	0.063
**BPS-CE-FB**	554.7(482.2-605.4)	540.0(405.9-597.9)	0.796
**BPS-OE-RL**	294.0(247.7-518.5)	421.3(366.0-545.4)	0.089
**BPS-OE-FB**	419.7(372.5-491.9)	434.9(356.0-538.9)	0.869

Abbreviations: Bi-Podalic Static (BPS); Open Eyes (OE); Closed Eyes (CE); Right-Left (RL); Forward-Backward (FB).

Table S2. Body sway values measured during dynamic upright standing in ASD and TD. Median values (interquartile range) are reported.

	ASD				TD			
**Variable**	1st	2nd	3rd	Overall p-values	1st	2nd	3rd	Overall p-values
**BPD-OE-RL**	35.9(29.6-43.9)	26.7(21.8-34.7)	28.2(25.9-30.5) §	0.020	27.9(19.7-45.2)	29.7(18.8-33.7)	26.1(15.6-32.5)	0.301
**BPD-CE-RL**	46.4(31.4-53.7)	32.5(25.0-39.0) §§	31.3(27.6-48.6)	0.006	45.9(41.6-53.2)	36.8(21.3-46.2)	36.6(18.8-41.3) *	0.020
**BPD-OE-FB**	110.7(86.0-133.4)	100.2(74.5-137.8)	66.6(53.0-89.9) §§§	0.027	62.2(44.9-104.2)	62.8(45.3-81.2)	56.9(46.2-68.2)	0.407
**BPD-CE-FB**	123.9(94.4-142.8)	97.5(66.6-128.9)	99.6(65.7-138.5)	0.497	152.2(92.7-197.7)	93.6(56.8-176.8)	72.3(39.0-131.5) **	0.045

Abbreviations: Bi-Podalic-Dynamic (BPD); Open Eyes (OE); Closed Eyes (CE); Right-Left (RL); Forward-Backward (FB); 1st External Perturbation; 2nd External Perturbation; 3rd External Perturbation. §Significant contrast 1st vs. 3rd (P=0.007); §§ Significant contrast 1st vs. 2nd (P=0.002); §§§Significant contrast 1st vs. 3rd (P=0.007); *Significant contrast 1st vs. 3rd (P=0.007); **Significant contrast 1st vs. 3rd (P=0.014).

Table S3. Surface electromyographic (sEMG) activity of leg muscles synchronized with body sway during static upright standing. Median values (interquartile range) are reported.

Variables	ASD	TD	p-Values
		**BPS-CE**	
**VL**	2.8(2.6-6.9)	3.4(2.8-13.2)	0.481
**BF**	5.8(3.1-12.0)	12.5(7.4-15.9)	0.105
**TA**	3.6(3.4-9.4)	3.7(3.3-4.6)	0.967
**LG**	5.2(3.7-6.3)	9.4(6.8-15.0)	**0.029***
		**BPS-OE**	
**VL**	2.9(2.6-4.0)	2.9(2.7-13.6)	0.481
**BF**	4.6(2.7-15.1)	10.9(4.7-19.6)	0.160
**TA**	3.6(3.4-12.2)	5.0(4.1-10.5)	0.393
**LG**	4.0(3.1-5.2)	7.5(5.0-14.4)	**0.011***

Abbreviations: Bi-Podalic Static (BPS); Open Eyes (OE); Closed Eyes (CE); Vastus Lateralis (VL); Biceps Femoris (BF); Tibialis Anterior (TA); Lateralis Gastrocnemius (LG); *Significant contrast ASD vs. TD ($P<0.05$)

Table S4. Surface electromyographic (sEMG) activity of leg muscles synchronized with body sway during dynamic upright standing. Median values and (interquartile range) are reported.

	ASD			Overall p-values	TD			Overall p-values
**BPD-OE**	1st	2nd	3rd		1st	2nd	3rd	
**VL**	7.0(2.7-18.3)	3.1(2.8-5.7)	2.8(2.7-7.1)	0.614	3.8(3.0-8.2)	3.4(3.0-5.4)	2.9(2.8-6.9)	0.497
**BF**	8.5(7.0-12.6)	9.5(7.2-11.2)	7.4(5.5-9.6)	0.407	17.6(10.3-28.5)	14.2(8.9-24.9)	17.7(7.6-28.4)	0.584
**TA**	7.6(3.5-38.3)	5.6(3.8-14.3)	4.1(3.5-18.7)	0.717	4.8(3.4-13.8)	3.7(3.1-8.0) ##	4.2(3.2-5.7) #	0.007
**LG**	14.4(6.2-44.8)	10.2(5.2-13.1)	8.1(4.4-17.2) §	0.008	14.2(8.3-34.4)	7.9(5.2-17.5)	14.2(8.1-25.4)	0.061
**BPD-CE**								
**VL**	7.5(2.8-28.8)	3.0(2.8-14.8)	2.9(2.5-10.6)	0.497	10.6(5.4-22.4)	9.4(5.3-28.9)	4.8(3.3-36.7)	0.202
**BF**	14.8(5.0-16.1)	13.2(4.0-22.0)	14.8(4.3-17.5)	0.905	20.8(17.2-46.7)	16.6(13.4-50.5)	19.1(9.5-40.8)	0.082
**TA**	7.6(3.9-56.2)	4.3(3.4-6.3)	3.8(3.6-6.6) *	0.027	5.5(3.1-19.4)	4.5(3.0-19.8)	4.0(2.9-11.1) +	0.006
**LG**	28.3(15.2-41.0)	14.8(10.1-32.3)	15.8(7.2-47.3)	0.407	24.6(15.9-42.5)	26.0(18.3-31.9)	20.3(7.9-34.0)	1.000

Abbreviations: Bi-Podalic-Dynamic (BPD); Open Eyes (OE); Closed Eyes (CE); Vastus Lateralis (VL); Biceps Femoris (BF); Tibialis Anterior (TA); Lateralis Gastrocnemius (LG); 1st External Perturbation; 2nd External Perturbation; 3rd External Perturbation. §Significant contrast 1st vs. 3rd (P=0.003); ##Significant contrast 1st vs. 2nd (P=0.004); #Significant contrast 1st vs. 3rd (P=0.014); *Significant contrast 1st vs. 3rd (P=0.007); +Significant contrast 1st vs. 3rd (P=0.002).
